# Annotations

**Published:** 1896-05-30

**Authors:** 


### ANNOTATIONS.

### The Amusements of Poor Women.

At the annual conference of the Women's Total Abstinence Union the other day Lady Elizabeth Biddulph insisted upon the imperative necessity for amusement and recreation which the women of the poorer classes experience. For many years past there has been an unceasing cry for amusements for working men. Not until quite lately has anybody thought of providing rational and recreative amusements for the household drudge and family slave. The difficulty now, as always, is a practical one. It is of no use whatever providing amusements which do not amuse. When the case of men is under consideration there seems to be no obstacle. There is always some member of the sterner sex who knows exactly what it is his fellows want in the amusement way, and is always ready with half a dozen different proposals. With women it is totally different. Notwithstanding that women are always telling us what they could do if men would but stand out of the way, we do not find a single practical proposal on behalf of women's amusements emerging from any woman's mind, great or small. Even Lady Elizabeth Biddulph herself, who sees so clearly how distressingly monotonous the lives of poor women in the small houses and mean streets of large towns must be, has nothing at all of a practical kind to propose. All that she says amounts only to this, that we must not be in too great a hurry to close the doors of the public-houses against poor women—at any rate, not until something better in the way of amusements is provided. In speaking thus we have no wish to bring railing accusations against the less inventive sex. Our object is, by setting the plain truth plainly before them, to provoke them to make some effort of a really practical kind towards the brightening and cheering of the lives of their "sisters in the slums."

### The Grindings of Civilisation's Mill.

No person unaccustomed to the study of blue books and annual reports would ever suspect what large numbers of matters of varied interest are often embedded therein, and what fascinating vistas of thought and speculation are opened out thereby. For example, the medical officer of health for the borough of Halifax, Dr. Ainley, has sent us, as is usual, his annual report for 1895; and on page 14 of the same he publishes a "table" with the heading "Analysis of Refuse Collected in the Borough of Halifax for the year 1895." This "analysis" is a very striking record. In the first place the total population of the borough is considerably under 100,000 persons, whilst the total refuse amounts to 49,605 loads. A "load" is no doubt a cart-load, that is, a sanitary cart-load. The ordinary deep sanitary cart of English boroughs is constructed to contain not less than two tons. Here, then, in the borough of Halifax, we find that for every single man, woman, and child grinding in the mill of civilisation there is a product in waste material amounting to not less than a ton per head per annum. For persons not accustomed to thinking in "tons" we may mention that a ton is the equivalent of 2,240 pounds. Therefore every man, woman, and child in Halifax reduces to waste, and in most cases to dangerous waste, a little more than six pounds of solid material every day. When we begin to go into detail, and to consider the kind of matter thus reduced to waste, we see how necessary it is for civilisation to be a stern disciplinarian in matters sanitary. For instance, among other waste matters, there were no less than 320 loads, or 640 tons of "garbage from fried fish shops" removed in Halifax during 1895. What maws those Yorkshiremen must have for fried fish! The "sweepings gathered from the streets and refuse from gullies" reached the total of 4,926 loads, or 9,852 tons; whilst the "droppings from horses" amounted to no less than 500 tons. All these are matters poisonous to human and animal life; as, indeed, were the whole of the 99,210 tons of waste matter removed. This little "table" gives us one small item of the labour, the imperative and Titanic labour, which civilisation is compelled to undertake merely to prevent itself being choked or poisoned by the waste and *débris* resulting from its own activity. The glimpse gives little trouble, and is well worth the making. The lesson it enforces is one of prompt and absolute obedience on the part of every human being among us to the minutest details of the sanitary laws which science and experience have so intelligently and competently framed for the protection of life and health.

### Ice Creams in Society and in the Streets.

From time to time in recent years paragraphs have gone the rounds describing the vileness of the compounds which are sold in the streets under the name of ice creams. These popular delicacies have been analysed, and people have been shocked to hear of the millions of microbes which have been found in every mouthful. Society folded its hands in holy horror, and declared that, even if only to protect the children who were the chief consumers of these dirty dainties, the trade in cheap ice creams must be put under restraint. And in fact there was much in favour of the contention. In 1894 the medical officer of health for Islington reported upon the ice cream industry in his district, noticing the dirty condition of the clothes and persons engaged in it, and the unsuitable nature of the premises. Dr. Klein also made a bacteriological investigation, showing the presence of enormous numbers of various bacteria including quantities of the *bacillus coli*, which from its normal habitat being the intestines of men and animals was taken to show a very offensive form of pollution. In 1895 a report on the subject was made for the British Institute of Preventive Medicine, in which it was shown, among other things, that street ice creams contained from 625,000 to 7,132,000 per cubic centimetre. From a paper recently read before the Society of Medical Officers of Health it would appear, however, that the street vendors are not the only people whose ice creams are crowded with living bacteria. The author, Mr. Nield-Cook, having investigated the ice creams of the streets, turned next to the ice creams of society, and with the object of discovering the bacteriological condition of the best which were procurable visited the shop of a well-known West-end confectioner, and bought a strawberry ice cream. Sad to relate this society ice cream was even worse than those of the Italian vendors in the streets, for it contained from 8,000,000 to 14,280,000 bacteria per cubic centimetre, and among them the same *bacillus coli*, of evil reputation, as before. This is a *tu quoque* with a vengeance. Perhaps the microbes in society ices come from the milk and cream, while those in the street ices come from many other sources as well. But both of them must be looked on with grave suspicion so long as they contain bacterial proof of pollution with such foul matters as are commonly associated with the *bacilli coli*.

